# Comparing the Impact of Tetrahydrocannabinol on 30‐Day Readmission Rates and Hospital Length of Stay in Patients With Depression

**DOI:** 10.1002/brb3.71792

**Published:** 2026-09-23

**Authors:** Mohammed E. Khedr, Yunjin Lee, Muhammed A. Mirza, Humberto Jimenez, Daniel Greer

**Affiliations:** ^1^ Ernest Mario School of Pharmacy Piscataway New Jersey USA; ^2^ St. Joseph's University Medical Center Paterson New Jersey USA

## Abstract

**Background and Objectives:**

Delta‐9‐tetrahydrocannabinol (THC), the psychoactive constituent of cannabis, continues to be critically evaluated for its potential therapeutic efficacy and associated safety concerns in major depressive disorder (MDD). Length of stay and readmission rates are key indicators of treatment response and prognosis. With rising cannabis use, understanding the effects of THC is essential. The objective of this study is to evaluate THC's impact on 30‐day readmission and length of stay among inpatient MDD individuals at an academic hospital.

**Methods:**

A single‐center retrospective chart review evaluated adult patients admitted for MDD between August 2018 and May 2025. Patients were categorized by positive or negative THC urine drug screen on admission. The primary outcomes were 30‐day psychiatric readmission and hospital length of stay. Unadjusted and adjusted analyses were performed. Logistic regression was used to evaluate 30‐day readmission, and multiple linear regression was used to evaluate length of stay. Adjusted models included age, gender at birth, psychiatric comorbidities, and polysubstance use.

**Results:**

Among 743 cases, 211 (28.4%) were THC positive. A THC‐positive urine drug screen was not independently associated with 30‐day psychiatric readmission (adjusted odds ratio [OR]: 0.821; 95% CI 0.319–2.115; *p* = 0.683) or difference in hospital length of stay (mean difference −0.242 days; 95% CI −0.983 to 0.499; *p* = 0.521). Older age was associated with longer length of stay, while female gender at birth was associated with shorter length of stay.

**Discussion and Conclusions:**

THC‐positive urine drug screens were not independently associated with 30‐day psychiatric readmission or differences in hospital length of stay among patients hospitalized for MDD. Larger studies are needed to further evaluate the relationship between cannabis use and psychiatric hospitalization outcomes.

## Graphical abstract

Among patients hospitalized for major depressive disorder, a positive THC urine drug screen was not independently associated with 30‐day psychiatric readmission or hospital length of stay. These findings suggest that patient factors beyond THC exposure may have a greater influence on hospitalization outcomes.

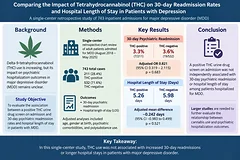

## 1 Introduction

Major depressive disorder (MDD) is a highly prevalent psychiatric condition characterized by a persistent low mood, lack of energy, and anhedonia. MDD was the third leading cause of global burden of disease in 2008, considering indicators such as the years affected with disease, disability, and financial costs, and affected 8.3% of US adults in 2021. MDD develops into multifaceted complications, adversely affecting interpersonal relationships and exacerbating chronic comorbidities such as diabetes, hypertension, and coronary artery disease (National Institute of Mental Health n.d., National Alliance on Mental Illness n.d). Affected individuals also experience elevated risks of comorbid anxiety and premature mortality (Bains and Abdijadid 2025).

Delta‐9‐tetrahydrocannabinol (THC), the primary psychoactive compound in cannabis, has become a prominent topic of discussion in the medical community due to its potential therapeutic benefits, though concerns about its safety (i.e., long‐term effects and drug‐drug interactions) are still ongoing. This concern is further amplified by the legalization of THC in many states, which has significantly increased its accessibility. A study done by Fink et al. (2023) revealed that there was an increase in THC use across all states from 2008 to 2019; notably, states with recreational cannabis legalization laws had caused a greater increase in the prevalence of positive THC urine drug screen on admission to the emergency department by 2.9% compared to states without legalization laws. As THC use becomes more widespread, there is a growing need to understand how it might affect different psychiatric conditions, including MDD.

Depression has a complex pathophysiology, affecting many neurotransmitters and receptors such as serotonin, norepinephrine, and dopamine. Emerging evidence implicates the endocannabinoid system in the pathophysiology of depression. For instance, individuals with depression exhibit lower circulating levels of the two primary endocannabinoids, anandamide (AEA) and 2‐arachidonoyl glycerol (2‐AG), resulting in decreased interaction with cannabinoid type 1 (CB1) and type 2 (CB2) receptors (Bright and Akirav 2022). THC is directly related to depression by interacting as a partial agonist/antagonist of the CB1 and CB2 receptors in the brain, affecting mood and emotional regulation by modulating the GABAergic, glutamatergic, serotoninergic, and noradrenergic systems. It is associated with psychomotor delay, anxiety, poor sleep quality, deficits in working memory, and apathy, all of which can worsen or exacerbate depressive symptomatology (Langlois et al. 2021).

When examining patients with MDD, users of cannabis report a decrease in stress and anxiety, although the ultimate exacerbation of baseline symptoms of depression with chronic use is still in question (Cuttler et al. 2018). To examine the effects of THC on patients with depression, careful evaluation of inpatient courses and outcomes is necessary. Length of stay and readmission rates are significant indicators of treatment response and long‐term prognosis of MDD (Zhu et al. 2022).

Few studies elucidate the clinical outcomes and healthcare overutilization associated with THC use upon admission in patients with depression. Nevertheless, some research studies examine relevant endpoints in other psychiatric conditions. One such study by Madero et al. (2020) evaluates the dose of cannabis consumed before hospitalization and its impact on the Brief Psychiatric Rating Scale (BPRS), a scale to measure the severity of psychotic symptoms, as well as length of stay as a secondary endpoint. The individuals with cannabis use the week prior to admission showed a higher mean BPRS score of 62.9 (SD = 11.1) compared to non‐users with a mean BPRS score of 55.8 (SD = 16.1). In this study, there was no statistically significant association between the quantity of cannabis consumed and the length of inpatient stay. Although this study successfully identifies the possible exacerbation of symptoms by cannabis, the endpoint of BPRS is best aimed at evaluating the positive and negative symptoms associated with schizophrenia and not MDD (Madero et al. 2020). Another retrospective cohort study by Soler et al. (2021) examines patients primarily with other psychiatric illnesses. Specifically, the study included a total of 370 patients (including 28 patients with depression) in an involuntary care unit. The inclusion criteria for this study were not limited to patients with MDD and classified MDD along mental organic disorders (Soler et al. 2021). In effective care, Dhaliwal et al. (2025) emphasize the need to address readmission for the sake of patients’ emotional, physical, and financial well‐being. Considering the lack of holistic data that incorporates various significant clinical endpoints and the underrepresentation of patients with depression, further research is warranted. Our study aims to strictly focus on patients with MDD while also including length of stay to expand the understanding of whether worse psychiatric symptoms translate into increased hospitalizations.

The goal of this study is to examine whether positive THC on urine drug screens correlates with increased inpatient psychiatric length of stay and 30‐day readmission rates among patients with MDD at a large academic medical center.

## 2 Methods

This single‐center retrospective chart review included patients with MDD discharged from the inpatient psychiatric unit between August 2018 and May 2025. The inclusion criteria consisted of a diagnosis of MDD, urine drug screen results, prescription of at least one antidepressant during admission and discharge, inpatient psychiatric hospitalization for MDD, and age 18 years or older. Exclusion criteria included prescription of an antidepressant without an MDD diagnosis, hospitalization for non‐psychiatric reasons, diagnosis of bipolar disorder, schizophrenia, or schizoaffective disorder, as well as prisoners, pregnancy, traumatic brain injury, or stroke. The patients admitted who tested positive for THC on a urine drug screen are referred to as the THC‐positive group and the patients who tested negative for THC are referred to as the THC‐negative group. Collected data included demographics, admission ICD‐10 codes (F32.0–F32.9), psychiatric comorbidities, suicidal behaviors, antidepressant therapy, prior psychiatric hospitalizations, time to readmission, and readmission length of stay. The primary outcomes were 30‐day psychiatric readmission and hospital length of stay. Secondary outcomes included 6‐month and 1‐year readmission rates.

Baseline characteristics were summarized by THC status and reported as frequencies and percentages for categorical data and the mean and standard deviation for continuous variables. The primary endpoints were analyzed in unadjusted and adjusted regression models. Thirty‐day readmission, a binary outcome, was analyzed using logistic regression with MedCalc statistical software (MedCalc Software Ltd, 2026). Results of the logistic regression were reported as odds ratio (OR) with a 95% confidence interval (CI). The unadjusted model included THC status as the sole predictor. The adjusted model included THC status as the primary exposure and adjusted for age, gender at birth, psychiatric comorbidities, and polysubstance use.

Length of stay was compared between THC‐positive and THC‐negative groups using an independent‐samples *t*‐test for the unadjusted analysis and multiple linear regression for the adjusted analysis. The unadjusted model estimated the difference in length of stay between the THC‐positive and THC‐negative groups. The adjusted model included THC status as the primary exposure and the same covariates included in the adjusted readmission analysis. Results of the multiple linear regression models were reported as mean difference with a 95% CI.

Endpoints were analyzed by case rather than by individual patient, with each case defined by the patient's initial admission. Readmissions occurring more than 1 year after the initial admission were considered new cases and analyzed separately. Therefore, individual patients could contribute multiple cases to the analysis.

## 3 Results

A total of 1700 cases were observed between August 2018 and May 2025, with 743 of the cases meeting the inclusion criteria. Baseline characteristics are shown in Table 1. The mean age was 38 years and 25.6% were female (Table 1). Of the 743 cases, 211 (28.4%) tested positive for THC and 532 (71.6%) tested negative. Roughly half of the cases in the study presented with psychiatric comorbidities (380 of 743; 51.1%). Co‐occurring DSM‐5 diagnoses included in our study were alcohol abuse and dependence, generalized anxiety disorder, borderline personality disorder, anti‐social personality disorder, adjustment disorder, anorexia nervosa, OCD, body dysmorphic disorder, PTSD, nicotine dependence, and antisocial personality disorder. Polysubstance use was reported in 208 patients (28.0%), with cocaine and benzodiazepines being the most common substances.

**TABLE 1:** Baseline characteristics.

Baseline characteristics		
	Positive for THC on admission	Negative for THC on admission
**Number of cases**	211	532
**Age**	31.3 ± 12.8	41.3 ± 18.8
**Gender at birth—**female (%)	79 (37.4%)	111 (20.9%)
**Race** (no. %)		
White	76 (36.0%)	173 (32.5%)
Black	52 (24.6%)	79 (14.8%)
Hispanic	28 (13.3%)	97 (18.2%)
Asian	7 (3.3%)	22 (4.1%)
Mixed/other	48 (22.7%)	161 (30.3%)
**Co‐occurring DSM‐5 diagnoses** (no. %)		
Yes	152 (72.0%)	228 (42.9%)
No	59 (28.0%)	304 (57.1%)
**Urine toxicology** (no. %)	57 (27.0%)	152 (28.6%)
PCP	3	1
Opiates	3	14
Cocaine	26	56
Benzodiazepine	22	58
Barbiturates	1	8
Alcohol	13	51
Amphetamine	7	7

Among all cases, there were a total of 110 readmissions, including 26 (23.6%) within 30 days, 53 (48.2%) between 30 days and 6 months, and 31 (28.2%) between 6 months and 1 year. Seven (3.3%) cases from the THC‐positive group and 19 (3.6%) cases from the THC‐negative group were readmitted within 30 days (unadjusted OR: 0.927; 95% CI: 0.384–2.237; *p* = 0.865). In the adjusted logistic regression model, THC was not associated with 30‐day readmission (adjusted OR: 0.821; 95% CI: 0.319–2.115; *p* = 0.683). Adjusting for age, gender at birth, psychiatric comorbidities, and polysubstance use did not change the impact THC status exposure had on 30‐day readmissions. Six‐month readmissions in the THC group were 5.2% (*n* = 11) compared to 7.9% (*n* = 42) in the group without THC exposure (OR: 0.63; 95% CI: 0.32–1.26; *p* = 0.096), and 1‐year readmissions were 3.3% (*n* = 7) in the THC group and 4.5% (*n* = 24) in the comparator (OR: 0.70; 95% CI: 0.30–1.64; *p* = 0.204). Unadjusted primary and secondary outcomes are shown in Table 2.

**TABLE 2:** Unadjusted primary and secondary endpoints.

Unadjusted primary and secondary endpoints				
	Positive for THC on admission	Negative for THC on admission	Mean difference [95% CI]	*p*‐value
**Length of stay** (days)	5.26	5.98	−0.715 [−1.419 to −0.011]	*p* = 0.0466
	**Positive for THC on admission**	**Negative for THC on admission**	**OR [95% CI]**	***p*‐value**
**Number of cases readmitted within**				
**0–30 days** (no. %)	7 (3.3%)	19 (3.6%)	0.92 [0.38–2.22]	0.865
**> 30 days–6 months** (no. %)	11 (5.2%)	42 (7.9%)	0.63 [0.32–1.26]	0.096
**> 6 months–1 year** (no. %)	7 (3.3%)	24 (4.5%)	0.70 [0.30–1.64]	0.204

Length of stay was shorter in the THC‐positive group in the unadjusted analysis (5.26 vs. 5.98 days; mean difference −0.715 days; 95% CI −1.419 to −0.011; *p *= 0.0466). However, THC exposure was no longer associated with length of stay after adjusting for the covariables (mean difference = −0.242 days; 95% CI: −0.983 to 0.499; *p *= 0.521). Additionally, psychiatric comorbidities (mean difference = −0.364 days; 95% CI: −1.024 to 0.297; *p* = 0.280) and polysubstance use (mean difference = −0.666 days; 95% CI: −1.389 to 0.058; *p* = 0.071) were also not associated with length of stay. Older age was associated with longer length of stay, with each 1‐year increase in age associated with a 0.044‐day (1.056 h) increase in length of stay (95% CI: 0.025–0.062 days; *p* < 0.001). Gender at birth was also associated with length of stay, with female patients demonstrating a shorter length of stay (mean difference = −1.138 days; 95% CI: −1.776 to −0.501; *p* < 0.001). There were no missing data for demographic characteristics, urine drug screen results, or variables included in the primary outcome analyses; therefore, no imputation or other methods for handling missing data were required. Unadjusted and adjusted endpoints are shown in Table 3.

**TABLE 3:** Unadjusted and adjusted primary endpoints.

**Unadjusted and adjusted primary endpoints**				
**Characteristic**	**30‐day readmissions OR [95% CI]**	**30‐day readmissions *p*‐value**	**Mean difference LOS (days) [95% CI]**	**Mean difference LOS *p*‐value**
THC (unadjusted)	0.927 [0.384–2.237]^a^	*p* = 0.865^a^	−0.715 [−1.419 to −0.011]^b^	*p* = 0.0466^b^
THC (adjusted)	0.821 [0.319–2.115]^c^	*p* = 0.683^c^	−0.242 [−0.983 to 0.499]^d^	*p* = 0.521^d^
Age (per 1‐year increase)	1.008 [0.987–1.030]^c^	*p* = 0.452^c^	0.044 [0.025 to 0.062]^d^	*p* < 0.001^d^
Female sex	0.980 [0.437–2.194]^c^	*p* = 0.960^c^	−1.138 [−1.776 to −0.501]^d^	*p* < 0.001^d^
Psychiatric comorbidities	0.486 [0.203–1.160]^c^	*p* = 0.104^c^	−0.364 [−1.024 to 0.297]^d^	*p* = 0.280^d^
Polysubstance use	0.750 [0.297–1.891]^c^	*p* = 0.542^c^	−0.666 [−1.389 to 0.058]^d^	*p* = 0.071^d^

^a^ Analyzed through univariate logistic regression.

^b^ Analyzed through independent sample *t*‐test.

^c^ Analyzed through multivariate logistic regression.

^d^ Analyzed through multiple regression.

## 4 Discussion

In this study, the number of readmissions was insufficient to provide adequate statistical power to draw reliable conclusions about the effect of THC on readmission rates. This study represents an important preliminary, proof‐of‐concept evaluation of the association between THC exposure and inpatient outcomes in patients with MDD. In the unadjusted length of stay, THC‐positive admissions appeared to be shorter, but this no longer held after factoring in covariates such as age, gender at birth, psychiatric comorbidity, and polysubstance use. This suggests that patient factors beyond THC use impacted length of stay. In particular, older age and male gender at birth were associated with longer inpatient admission duration. The readmission analysis showed a similar relationship as the length of stay in that there was no independent association between THC use on admission and 30‐day readmission rates. The strengths of this study include a large real‐world sample size and inclusion of clinically relevant endpoints, including 30‐day, 6‐month, and 1‐year readmissions and length of stay.

Existing literature suggests a notable correlation between the use of cannabis and the prevalence of MDD. A systematic review by Onaemo et al. (2021), incorporating eight nationally representative epidemiological studies, reported the risk of comorbid cannabis use disorder with generalized anxiety disorder (pooled OR: 2.99) and comorbid cannabis use disorder with MDD (pooled OR: 3.22). These findings are further supported by a systematic review and meta‐analysis performed by Lev‐Ran et al. (2014), which evaluated 57 studies and demonstrated an increased risk of depression among cannabis users (pooled OR: 1.17; 95% CI 1.05–1.30), with a higher risk observed in heavy users (pooled OR: 1.62; 95% CI 1.21–2.16) compared with non‐users or light users. Additionally, a recent systematic review and meta‐analysis of 22 longitudinal studies found that cannabis use was associated with a 29% increased odds of developing depression over time (OR: 1.29, 95% CI 1.13–1.46), although the authors emphasized that the available evidence demonstrates an association rather than causation (Churchill et al. 2025). Collectively, these data suggest an association between THC exposure and the prevalence of MDD, highlighting the need for further research to elucidate its role in the development or exacerbation of depressive disorders.

Careful evaluation of inpatient courses and outcomes is necessary to examine the effects of THC on patients with depression. Endpoints such as length of stay and readmission rates are significant indicators of treatment response, healthcare utilization, and longer term prognosis in MDD. Additionally, the case‐based analytic approach allowed for the capture of repeated hospitalizations, better reflecting real‐world disease burden and healthcare utilization.

Limitations of this study include its retrospective single‐center design, reducing the generalizability of these results to a wider context. Potential confounding factors included polysubstance use, psychiatric comorbidities, gender at birth, age, and the lack of data on THC dose, formulation, or duration of use. Patients with polysubstance use were included to enhance the generalizability of the findings to real‐world populations. Future studies with larger cohorts should perform subgroup analyses to better characterize the influence of additional substance use on treatment outcomes. Additionally, patients with positive urine THC were not stratified by amount or frequency of use. As described by Lev‐Ran et al. (2014), heavy THC users displayed a higher OR for developing depression compared to non‐users or light‐THC users. This highlights the need for further research to identify the effects of the dose, formulation, and duration of THC use on MDD. An additional limitation is that analyses were conducted by case rather than by patient. Although readmissions occurring more than 1 year after the initial hospitalization were considered new cases to better reflect changes in patient characteristics, treatment, and clinical status over time, some individuals contributed multiple cases to the analysis. As a result, observations may not have been fully independent, and future studies should consider patient‐level analyses or statistical methods that account for repeated measures. This topic warrants further investigation as the legalization of marijuana in different states has increased its accessibility. Whether this increased accessibility affects hospitalization outcomes related to MDD remains to be established.

These findings highlight key feasibility considerations and emphasize the lack of potential clinical relevance of THC exposure in this population. As cannabis use continues to increase through expanding legalization across the country, understanding its impact on psychiatric outcomes is increasingly important. These results support the need for large, multi‐center studies with extended follow‐up periods and a more granular characterization of THC exposure (e.g., dose, frequency, and formulation). Future research should also aim to control for confounding variables such as polysubstance use and comorbid psychiatric conditions to better elucidate causal relationships and inform clinical decision‐making.

## Author Contributions

**Mohammed E. Khedr**, **Muhammed A. Mirza**, and **Yunjin Lee** conducted the data collection. All authors contributed to the study conception, manuscript preparation, and critical review.

## Funding

The authors have nothing to report.

## Ethics Statement

The study is IRB approved.

## Conflicts of Interest

The authors declare no conflicts of interest.

## Data Availability

The data are not publicly available due to privacy restrictions.
